# Molecular correlates of localized versus co-occurring chronic pain conditions

**DOI:** 10.1186/1744-8069-10-S1-O11

**Published:** 2014-12-15

**Authors:** Andrea G Nackley

**Affiliations:** 1 Center for Pain Research and Innovation, University of North Carolina, Chapel Hill, NC 27599-7455, USA

## Background

Complex chronic pain conditions, including temporomandibular disorder (TMD), vestibulodynia (VBD), irritable bowel syndrome (IBS), and widespread bodily pain (WBP), represent a significant healthcare problem. Current treatment regimens remain ineffective due to the conditions’ unclear etiology and heterogeneous clinical manifestation. An emerging literature suggests that localized pain conditions occurring in isolation result from local increases in peripheral afferent activity, while those occurring in concert result from central dysregulations in pain processing. To understand the nature of these complex conditions and improve standards of care, the identification of unique biological signatures and pathways that map onto distinguishing clinical features is required. Thus, the objective of this session is to discuss the relationship between pain, related psychological characteristics, cytokine levels, and microRNA expression profiles in individuals with localized versus co-occurring chronic pain conditions.

## Materials and methods

Two independent case-control studies were conducted. The first study included 344 participants: 103 with TMD, 66 with TMD+WBP, and 175 healthy controls. The second study included 78 participants: 33 with VBD, 23 with VBD+IBS, and 22 healthy controls. Both cohorts included assessments of experimental pressure and thermal heat pain (measured using an algometer and Peltier device, respectively), self-reported health and psychological phenotypes (measured using standardized questionnaires), and circulating cytokine protein levels (measured using a multiplex assay). The VBD cohort also included assessments of intracellular microRNAs (measured using OpenArray), which are small noncoding pieces of RNA that control gene expression.

## Results

Compared to individuals with localized pain and healthy controls, those with TMD+WBP or VBD+IBS reported decreased general and physical health; increased somatization; increased interference of pain on daily activities; and increased remote bodily pain. All cases demonstrated increased circulating levels of the pro-inflammatory cytokine interleukin-8. However those with TMD or VBD demonstrated a compensatory increase in the anti-inflammatory cytokine interleukin-1 receptor antagonist, while those with TMD+WBP or VBD+IBS did not. Individuals with VBD displayed dysregulation of 10 microRNAs that cumulatively regulate pathways vital for pain processing and estrogen signaling. Those with VBD+IBS displayed dysregulation of 11 microRNAs important for pain processing, cellular physiology, and insulin signaling. Finally, cytokine and microRNA expression profiles were correlated with pain-relevant intermediate phenotypes.

## Conclusions

Individuals with localized and co-occurring pain conditions differ with respect to clinical characteristics and molecular profiles, suggesting unique underlying pathologies that contribute to each subtype irrespective of the specific anatomic site(s) involved. Cytokines and microRNAs may represent valuable tools for differentiating between these subtypes.

